# Fibrillar Collagen Quantification With Curvelet Transform Based Computational Methods

**DOI:** 10.3389/fbioe.2020.00198

**Published:** 2020-04-21

**Authors:** Yuming Liu, Adib Keikhosravi, Carolyn A. Pehlke, Jeremy S. Bredfeldt, Matthew Dutson, Haixiang Liu, Guneet S. Mehta, Robert Claus, Akhil J. Patel, Matthew W. Conklin, David R. Inman, Paolo P. Provenzano, Eftychios Sifakis, Jignesh M. Patel, Kevin W. Eliceiri

**Affiliations:** ^1^Laboratory for Optical and Computational Instrumentation, University of Wisconsin–Madison, Madison, WI, United States; ^2^Department of Biomedical Engineering, University of Wisconsin–Madison, Madison, WI, United States; ^3^Department of Medical Physics, University of Wisconsin–Madison, Madison, WI, United States; ^4^Department of Computer Sciences, University of Wisconsin–Madison, Madison, WI, United States; ^5^Department of Cell and Regenerative Biology, University of Wisconsin–Madison, Madison, WI, United States; ^6^Department of Biomedical Engineering, University of Minnesota, Minneapolis, MN, United States; ^7^Morgridge Institute for Research, Madison, WI, United States

**Keywords:** tumor microenvironment, collagen organization, fibrillar collagen, curvelet transform, image analysis software, second harmonic generation microscopy, breast cancer, pancreatic cancer

## Abstract

Quantification of fibrillar collagen organization has given new insight into the possible role of collagen topology in many diseases and has also identified candidate image-based bio-markers in breast cancer and pancreatic cancer. We have been developing collagen quantification tools based on the curvelet transform (CT) algorithm and have demonstrated this to be a powerful multiscale image representation method due to its unique features in collagen image denoising and fiber edge enhancement. In this paper, we present our CT-based collagen quantification software platform with a focus on new features and also giving a detailed description of curvelet-based fiber representation. These new features include C++-based code optimization for fast individual fiber tracking, Java-based synthetic fiber generator module for method validation, automatic tumor boundary generation for fiber relative quantification, parallel computing for large-scale batch mode processing, region-of-interest analysis for user-specified quantification, and pre- and post-processing modules for individual fiber visualization. We present a validation of the tracking of individual fibers and fiber orientations by using synthesized fibers generated by the synthetic fiber generator. In addition, we provide a comparison of the fiber orientation calculation on pancreatic tissue images between our tool and three other quantitative approaches. Lastly, we demonstrate the use of our software tool for the automatic tumor boundary creation and the relative alignment quantification of collagen fibers in human breast cancer pathology images, as well as the alignment quantification of *in vivo* mouse xenograft breast cancer images.

## Introduction

Fibrillar collagen organization influences cell behavior and has been implicated in a wide array of diseases ranging from osteogenesis imperfecta (OI), muscular dystrophy, wound healing, diabetes, to a number of cancers (Keely et al., 1995a, b; Roskelley et al., 1995; Simian et al., 2001; Provenzano et al., 2006, 2008a; Ghajar and Bissell, 2008; Conklin et al., 2011; Drifka et al., 2015, 2016; Hanley et al., 2016; Wen et al., 2016). In particular, changes in collagen organization have been linked to cancer progression and stage in breast cancer (Conklin et al., 2011), ovarian kidney cancer (Best et al., 2019), pancreatic cancers (Drifka et al., 2015, 2016), and multiple other cancers (Hanley et al., 2016). Increased collagen alignment in breast and pancreatic cancer has also been correlated to poor patient prognosis. Specifically, non-invasive regions are contained by collagen fibers oriented parallel to the tumor boundary while regions of local invasion possess areas where collagen has been realigned perpendicular to the tumor boundary to facilitate local invasion (Provenzano et al., 2006). Therefore, it has been hypothesized that the angle of collagen fibers relative to the tumor boundary may be used as a predictor of imminent invasion and metastasis (Provenzano et al., 2009). These collagen changes, known as Tumor-Associated Collagen Signatures (TACS)_ENREF_4 (Provenzano et al., 2006), fall into three categories named TACS-1, TACS-2, and TACS-3, where TACS-3 fibers have a larger relative angle with respect to the tumor boundary than TACS-1 or TASC-2 fibers. Many recent, novel characterizations elucidating the relationship between collagen and disease state have arisen due to rapid development in the area of non-invasive high-resolution imaging methods, such as the label-free imaging method of second harmonic generation (SHG) microscopy (Stoller et al., 2002; Mohler et al., 2003), that can track spatial and temporal changes in the collagen matrix.

However, even with these great advances and improved ability to image collagen, there is still a dearth of robust and flexible computational methods for quantitative analysis of collagen architecture. To the best of our knowledge, there are only a few open-source tools currently available for collagen fiber quantification, with most focusing on orientation and alignment estimation including Fourier transform-based CytoSpectre (Kartasalo et al., 2015) and pixel-wise orientation-based ImageJ/Fiji (Schindelin et al., 2012; Schneider et al., 2012) plugins of OrientationJ (Rezakhaniha et al., 2012) and FibrilTool (Boudaoud et al., 2014). To track fiber orientation, the Fourier transform-based methods (Chaudhuri et al., 1987; Sander and Barocas, 2009; Kartasalo et al., 2015; Pijanka et al., 2019) rely on spatial frequencies in power spectrum analysis to detect the intensity changes at the fiber edges. They are insensitive to the image background noise and have been commonly used in the estimation of overall or dominant fiber orientation and anisotropy of orientations in an image or image tile. The main limitation of this approach is the lack of individual fiber information. The pixel-wise orientation-based methods (Rezakhaniha et al., 2012; Boudaoud et al., 2014; Püspöki et al., 2016) use derivative information and can estimate the orientation of individual pixels. However, additional strategies are needed to overcome the impact of image noises and to find the pixels relevant to fiber edges. Both tools do not provide direct individual fiber information.

To be noted, the collagen fibril has been defined in structural terms as semi-crystalline aggregates of collagen molecules and their crosslinks. Where several of these fibrils, sometimes called “thin fibrils”, are bundled together with associated molecules to form much larger fibers, they have been termed “fibril bundles.” Although it is vitally important to differentiate thin fibrils and fibril bundles to understand how certain molecular associations occur with collagen fibers, this is not the focus of this study. We use the terms “fibril” and “fiber” interchangeably in this paper.

Curvelet transform (CT) (Candes and Donoho, 2000; Candès and Donoho, 2004; Candès et al., 2006) has the unique features for image denoising and edge enhancement. Moreover, curvelets can directly provide an optimal sparse representation of the collagen image and thus can track individual fibers and fiber branches either they are straight or curvy. We have put significant efforts on the development of two open-source CT-based collagen quantification tools: CurveAlign (Schneider et al., 2013; Bredfeldt et al., 2014a; Liu et al., 2017) and CT-FIRE (fiber extraction) (Bredfeldt et al., 2014b). These MATLAB (MathWorks, Natick, MA) tools were designed to meet user-defined features in terms of accuracy, speed, and compatibility. Our tools can do both curvelet-based fiber orientation and location representation and individual fiber tracking. They support image-based, region of interest (ROI)-based, or fiber-based quantification of collagen features including geometry properties, density, alignment, and relative alignment. They also support fast computing using multi-core or distributed computer clusters for large datasets and fast individual fiber estimation using optimized C++ MEX^1^ files for potential real-time applications. Our tools have been utilized for a wide range of applications including collagen quantification in breast cancer (Corsa et al., 2016; Conklin et al., 2018), pancreatic cancer (Drifka et al., 2015), renal cancer (Best et al., 2019), cervical tissue (Reusch et al., 2010), cardiac disease (Kouris et al., 2011), wound healing (LeBert et al., 2015; Israel et al., 2017), as well as quantification of filamentous structures such as microtubules (Heck et al., 2012).

CurveAlign was developed first for bulk collagen alignment studies and had the main goal of quantifying all fiber angles within a ROI relative to a user-defined boundary be it a straight line or a tumor boundary. In this approach, curvelets can be used to robustly detect and represent collagen fibers including their locations and orientations in noisy and complex conditions, on which numerous collagen metrics or features can be built. As our research grew in investigating the role of collagen in cancer progression and invasion, we wanted to investigate how individual fiber parameters could influence cancer and other diseases. Out of this need came the development of CT-FIRE to analyze individual fiber metrics such as length, width, angle, and curvature using CT as a pre-processing step to reduce noises and enhance fiber edges. In addition to boundary-based relative angle quantification, the current version of CurveAlign can be used to extract other collagen fiber features, such as localized fiber density, fiber alignment (i.e., anisotropy), and the spatial relationship between fiber and the associated boundary. In addition, the extracted individual fibers extracted by CT-FIRE can be imported into the CurveAlign for additional feature extraction mentioned above.

We have previously reported the general framework and basic concept of both CT-FIRE (Bredfeldt et al., 2014b) and CurveAlign (Bredfeldt et al., 2014a) as well as the protocol of using CurveAlign for fibrillar collagen alignment quantification (Liu et al., 2017). In this paper, we present an introduction to the curvelets fiber representation with detailed description and validation that has not been previously published as well as some latest developments in both tools. Specifically, we will first introduce the idea of CT and the optimal fiber representation with curvelets in CurveAlign, then we will introduce new features and modules that are available to CT-FIRE and CurveAlign users, including (1) fast individual fiber tracing with C++-based code optimization to speed up individual fiber tracking; (2) Java-based synthetic fiber generator to generate synthetic fibers according to user-specified fiber parameters for methods validation and synthetic image datasets creation; (3) automatic registration and tumor boundary creation using bright-field hematoxylin and eosin (H&E) and SHG images; (4) parallel computing for batch-mode analysis using either a single multi-core computer or distributed computers; (5) ROI analysis to analyze user-defined or program-computed regions; (6) complementary pre- and post-processing modules including individual fiber visualization and thresholds-based fiber selection, output file combination, and others. We also provide a validation of the fundamental features of our tools, i.e., the tracking of individual fibers and fiber orientations, using synthesized fibers generated by the synthetic fiber generator mentioned above. We then compared the orientation calculation on real SHG images of pancreatic tissue samples between our method and three other quantitative methods including manual measurement, OrientationJ, and CytoSpectre. Lastly, we demonstrate the automatic boundary creation and the quantification of collagen fiber organization in TACS features in breast cancer as well as the alignment quantification of *in vivo* mouse xenograft breast cancer images.

## Materials and Equipment

To validate our tools’ capability of individual fiber tracking and fiber representation for both straight fibers and curvy fibers, we generated two datasets of 100 synthetic fiber images with different straightness using synthetic fiber generator (version 1.1) that is described in detail in Section “Synthetic Fiber Generation Using User-Specified Fiber Parameters.” The fibers of each image in the first dataset are straight (i.e., straightness equals 1) while the fibers in another dataset have a straightness of 0.92. The other parameters were set as follows: image size: 512 × 512 pixels; number of fibers in each image: 30; alignment coefficient: 0.2; mean angle: 90°; fiber length: 60 pixels; fiber width: 5.0 pixels; edge buffer: 10 pixels; image noise level: 50; three smoothing methods offered by the fiber generator were selected using the default setting values for each smoothing. Other parameters not mentioned here were kept as their default values.

To demonstrate the use on real biomedical images, we used human pancreatic tissues of normal stroma and tumor grades 1–3 to show the orientation calculation. In addition, we used a human breast cancer tumor microarray (TMA) core identified as TASC-3 positive to show the boundary creation and relative angle measurement. Moreover, we used an image from a mouse breast cancer model to show the quantification of alignment at different regions. The SHG images for both breast sample and pancreatic sample were acquired on our home-built SHG microscope and were described in previous publications (Bredfeldt et al., 2014a; Drifka et al., 2015) while the bright-field image of the breast sample was acquired on an Aperio CS2 scanner system (Leica Biosystems, Buffalo Grove, IL, United States) at the Translational Research Initiatives in Pathology Laboratory at the University of Wisconsin Madison. In the mouse breast cancer model, a xenograft of MDA-231 breast cancer cells was implanted for 3 weeks in an adult mouse using methods similar to those described in Provenzano et al. (2008b). The SHG image used here shows the areas near the graft boundary. It was acquired on a Prairie Ultima IV multiphoton microscope (Bruker Technologies, Middleton, WI) with a Olympus 40 × /0.8 water dipping objective (Olympus, Center Valley, PA).

To be noted, the 100 synthetic straight fiber images and the human pancreatic tissue images were also used in a speed test on the MEX functions (Supplementary Figure 1).

## Methods

### Curvelet Transform

The CT, which was originally proposed in Candes and Donoho (2000) is an overcomplete representation in terms of local, wavelet-like functions, each associated with a specific scale, orientation, and position. It is shown that the m-term approximation using a curvelet representation outperforms both the Fourier and wavelet representations, in the sense that fewer curvelets, than sinusoids or wavelets, are needed to accurately represent image structure. Conceptually, the CT is a multiscale pyramid with many orientations and positions at each length scale and needle-shaped elements at fine scales. Curvelets obey a parabolic scaling relation that says that at scale 2^−*j*^, each element has an envelope that is aligned along a “ridge” of length 2^−*j*/2^ and width 2^−*j*^ (Candès et al., 2006). A brief overview of the mathematical framework from Candès et al. is now presented to give the reader a formal representation of curvelets. Consider the case where our spatial variable *x* lies in *ℝ*^2^. Start with a windowing of the space both radially and angularly and call these windows *W*(*r*) and *V*(*t*), respectively, where *r* ∈ (1/2, 2)) and *t* ∈ [−1, 1]. Now, consider the frequency window *U*_*j*_ defined in the Fourier domain by:

(1) $$U_{j\,\left(r,\theta \right)}=2^{-3\,j/4}\,W\,\left(2^{-j}\,r\right)\,V\,\left(\frac{2^{\left\lfloor j/2\right\rfloor }\,\theta }{2\,\pi }\right)$$

Where ⌊*j*/2⌋ is the integer part of *j*/2. Therefore, the support of *U*_*j*_ is a polar “wedge” defined by the support of *W* and *V*. The equispaced sequence of rotation angles are defined as $\theta_{l}=2\,\pi \cdot \,2^{\left\lfloor -\frac{j}{2}\right\rfloor }\cdot l$ with *l* = 0, 1, 2,… such that 0≤ θ_*l*_ < 2π, with translation parameters *k* = (*k*_1_,k_2_) ∈ *ℤ*^2^. The waveform *φ*_*j*_(*x*) is defined by its Fourier transform, ${\hat{\varphi }}_{j}\,\left(\omega \right)=U_{j}\,\left(\omega \right)$. Curvelets at scale 2^−*j*/2^, orientation θ_*l*_, and position $x_{k}^{j,l}=R_{\theta_{l}}^{-1}\,\,\left(k_{1}\cdot \,2^{-j},k_{2}\cdot \,2^{-j/2}\right)$ where *R*_θ_ is the rotation by θ radians, can now be defined as:

(2) $$\varphi_{j,k,l}\,\left(x\right)=\varphi_{j}\,\left(R_{\theta_{l}}\,\left(x-x_{k}^{j,l}\right)\right)$$

A curvelet coefficient is then the inner product between an element *f* ∈L^2^(*ℝ*^2^) and a curvelet *φ*_*j*,*k*,*l*_:

(3) $$c\;\left(j,k,l\right)\;\;:=\;\langle \text{f,}\;\varphi_{j,k,l}\rangle \;=\;\int_{{\mathbb{R}}^{2}}\text{f}\left(\text{x}\right)\;\bar{\varphi_{j.k.l}\left(\text{x}\right)\text{dx}}$$

### Optimal Fiber Representation and Analysis With CurveAlign CT Mode

Curvelets in CT can optimally represent line-like structures in a sparse manner at different scales, orientations and locations. In CurveAlign, we implemented a fiber analysis mode called CT mode to trace the representative fiber orientations and then do corresponding analysis. Some key steps in the implementation are as follows:

(1) Perform a 2D FDCT (through wrapping in MATLAB) on the input image;
(2) Select the scale of interest (the second finest scale by default) and discard the CT coefficients in other scales;
(3) Threshold the remaining coefficients based on a user-defined threshold (generally keeping only the largest 0.1–1%);
(4) Find the center and spatial orientation of each curvelet corresponding to the remaining coefficients;
(5) Group the adjacent curvelets within a given radius to estimate local fiber orientations; and
(6) Perform application-specific analytics using the measured angles and locations.

CT mode owns the same analysis modules available to CT-FIRE individual fiber analysis mode described in Bredfeldt et al. (2014a) where the CT mode uses grouped curvelets to estimate fiber orientations while the CT-FIRE mode uses the information from individual fibers. CurveAlign CT mode has a boundary-free measurement scheme and a boundary-enabled measurement scheme, with the former measuring the distribution of fiber alignment in an image with respect to an absolute reference and the latter measuring the distribution of fiber alignment in an image relative to a user-defined or automatically computed boundary. Once the angles have been determined, further statistics are computed, for example, the mean, median, variance, standard deviation, skewness, and kurtosis of the distribution are calculated and written out to a CSV file. The directional statistics methods (Berens, 2009) are used for all statistical analysis of the fiber orientation information. The overall alignment of the fibers is determined by calculating the resultant vector length of all orientation vectors. This yields a unitless number between 0 and 1 that indicates how well the distribution is grouped around the median angle, with 0 being completely random and 1 being completely aligned in the direction of the median angle. In both of the measurement schemes described above, there is no need for any pre-processing such as thresholding or denoising. Both are accomplished by the selection of the appropriate curvelet coefficients for the analysis. The very highest and lowest scales of the CT, which contain the high-frequency noise and the low-frequency background in the image should be discarded.

The main available outputs from CurveAlign CT mode are as follows: (1) Overlay Image—This allows the user to see where curvelets were found within the image by overlaying center point and orientation of each curvelet on the original image; (2) Local Orientation Map—Indicates localized alignment with respect to the boundary or to other nearest fibers regions within an image; (3) Reconstructed Image—this is an image reconstructed from the remaining curvelets. It shows all of the edges in the image that were measured; (4) Histogram Plot—a bar histogram of the measured angles, with respect to either the boundary or the horizontal axis if no boundary is used; (5) Compass Plot—an angular histogram of the measured angles; (6) Values List—the values of the measured angles as well as basic descriptive statistics; and (7) Features list include all the localized density and alignment values for each curvelet. Image outputs are saved in tiff format. Histograms, compass plots, data values, and statistics are saved in CSV files.

### Fast Individual Fiber Estimation With C++ Code Conversion

The fiber extraction algorithm (or FIRE) (Stein et al., 2008) is part of the CT-FIRE (Bredfeldt et al., 2014b) software tool for individual fiber extraction. It may take a couple of minutes for this algorithm to process an image with the size of 512 × 512 pixels using, for example, a Windows computer with 1.9 and 2.5 GHz Intel dual-core and 16 GB memory. This processing speed would not allow for a real-time analysis. Our code performance evaluation showed that the main reason is due to inefficient loop operations in MATLAB. MATLAB offers some strategies such as data vectorization, which can save computational time but is limited by the data structure currently used in the FIRE algorithm. Hence, in order to significantly speed up the fiber extraction, we converted three major steps of the FIRE algorithm from MATLAB code to C++ MEX code, part of which is using the C++ multithread library (i.e., OpenMP) for parallel processing. The C++ MEX implementation mostly follows the algorithm originally described but with some exceptions. Some key points of the implementation for each of the three steps are described below.

#### Find Nucleation Points

Nucleation points follow the same definition described in FIRE (Stein et al., 2008); i.e., they occur at local maxima (of a box whose size is defined by a pre-defined radius) where the distance function also exceeds a pre-defined threshold. The search for nucleation points loops through all the pixels with assessment of relevant local box of each pixel, with the loop in one dimension being parallelized. The thread number is used as index to the nucleation point. Given the same input, this implementation may not necessarily yield the same output as the MATLAB code because the C++ MEX function uses a different random number generator to randomize the distances.

#### Extend From Nucleation Points to Form Fiber Branches

In this conversion, both the structure of a fiber and the function of extending branches from nucleation points are defined. The procedure of extending branches at each nucleation point follows the original algorithm but how to remove the redundant directions is different. The extension at each nucleation point is independent and is parallelized. At each nucleation point, there are mainly three steps:

(1) Look for the local maximum points (LMPs) as the extension points, which occur on the surface of a box (whose size is defined by the distance value of the current position) and their distance values are larger than a pre-defined threshold. If two LMPs are too close, only one LMP is kept;
(2) When a fiber segment is extended from one point to another, extension direction is calculated and the fiber tip direction is updated with memory on previous directions; and
(3) If there is no LMP or another nucleation point is found, the branch extension will be terminated. Thereafter, the parallel processing is used to populate the fiber link map, delete duplicated fibers, and populate fiber index map.

Given the fact that this C++ implementation does not exactly follow the same procedure as the MATLAB implementation, the two implementations may yield slightly different extended branches for a same set of nucleation points.

#### Link Fiber Branches to Form a Fiber

This conducts fiber linking when both branches and fiber segments share a same nucleation point. The linking criterion is first found first link. In other words, if a pair of branches are linked, they will be removed from the branches pool until all the branches are looped through. To link two branches, they have to share the same nucleation point, and the directions of the branch tips have to be similar or within the threshold of angle differences. If more than one branches satisfy the angle and nucleation joint requirement, the one that is more aligned to the current branch (or has the least difference in direction) is selected. In the calculation of fiber end direction, a spacing parameter is used to specify the distance between the end and another vertex along the fiber.

In Supplementary Figure 1, a speed test on both synthetic images and real images shows that MEX functions are more than 100x faster than the original code to complete the above three steps of fiber extraction from single images while leading to similar orientation and alignment. This is a first step toward greatly improving speed of the CT-FIRE program, and future directions will build on this work and address other performance bottlenecks in the collagen analysis workflow.

### Synthetic Fiber Generation Using User-Specified Fiber Parameters

To verify the accuracy and test the limits of CT-FIRE and CurveAlign, a Java-based software module was developed and can be launched from CT-FIRE that allows the user to generate synthetic images of collagen fibers with specific, known properties.

To generate fibers, a length, starting width, and straightness are all drawn from the user-defined distributions. The end-to-end directions are chosen such that all fibers, when considered together, have the specified mean angle and alignment. A recursive bridging algorithm is used to choose a specific path between fiber endpoints. Suppose that a fiber consists of 16 unit length segments, and that it has endpoints at (0, 0) and (10, 0). A midpoint is chosen by randomly sampling from the intersection of two disks of radius 8 centered at the endpoints. These disks represent the region reachable from an endpoint after 8 steps. The disk intersection process is repeated between the newly chosen midpoint and each of the endpoints in turn. The process continues recursively until all points composing the fiber have been determined. The width of the fiber changes from segment to segment by adding a uniformly distributed delta. This delta is specified in the user interface as “width change.” The main features of this fiber generator include the following:

(1) The definition of all fiber parameters follows those in CT-FIRE and CurveAlign. This makes it possible to validate our existing tools while the output can be used to generate new fiber images.
(2) The number of fibers, fiber width change, length of fiber segments (each fiber is composed of linear segments), alignment of all fibers, and mean orientation of all fibers can be specified.
(3) The distribution of length, width, and straightness can be set as Gaussian, uniform, or piecewise linear. The Gaussian distribution is parameterized by its mean and standard deviation, the uniform distribution by its minimum and maximum values, and the piecewise linear function by comma-separated lists of two dimensional coordinates, i.e., *X* and *Y* values.
(4) Three different smoothing methods were implemented and can be used independently or together. The smoothing methods include the following: (1) bubble: sweeps over the fiber and swaps adjacent segments; the parameter value gives the number of passes to make; (2) swap: swaps random pairs of segments; the parameter value gives the average number of swaps attempted per segment; and (3) spline: uses polynomial splines to interpolate extra points; the parameter value is roughly the ratio of the number of points after smoothing to the number of points before smoothing.
(5) Image width and height as well as the size of the edge buff are required for the synthetic fiber image generation.
(6) To mimic real images, additional operations can be applied to the output image. These include rescaling, down sampling, blur, addition of noise, distance filter, thresholding, and normalization.

### Automatic Tumor Boundary Creation

To have a map of tumor locations for relative alignment analysis, besides a manual boundary creation function, we developed an automatic boundary creation module based on the bright-field H&E image. This module can be used to first register the H&E image to the SHG image, and then do a subsequent segmentation. In the registration algorithm: first enhance the colors using decorrelation stretch (Gillespie et al., 1986), then extract the collagen-like structure from bright-filed image by color separation, and lastly register the image from the previous step to the SHG image using an iterative algorithm at different scales. More details about this registration algorithm can be seen in our recent work (Keikhosravi et al., 2019). The segmentation algorithm includes the steps of image color enhancement, color segmentation, finding nuclei and grouping them, and removing unwanted regions. To improve the accuracy of the segmentation, a white balancing as a pre-processing step was implemented. Two steps of color enhancement in the algorithm were implemented to overcome the variations in stain hue or imaging systems.

To best use the registration algorithm, the fields of view of the bright-field and SHG images shouldn’t differ by, e.g., more than 10% and the SHG image should show detectable collagen signals. To reduce the errors introduced by possible subjective selection of parameters, the user only needs to enter the pixel resolution of the SHG image (i.e., pixel per micron one parameter) to run this module. This module can process a single image pair or multiple ones sequentially. The output of this module are tumor boundary masks following the mask naming convention and path requirement in CurveAlign.

### Parallel Computing With Multi-Core Computers or Distributed Computers

To speed up the batch-mode analysis for processing, e.g., hundreds even thousands of images, we implemented parallel processing/computing in two ways:

#### Utilization of Multi-Core Processing

Parallel loops were implemented for CT-FIRE fiber extraction, CT-FIRE post-processing, and CurveAlign full image analysis. If all the cores are used simultaneously, using parallel loops can reduce the computation time to about 1/*n* (number of cores) of the normal process time when the tool runs on a single core. In the implementation, we make sure that all the processes in the parallel loop are independent, the functions that are not supported in parallel computing are replaced, and the random number generator generates the same numbers that are to be used in the algorithm such that the results from the parallel computing are identical to the conventional sequential processing. To enable the parallel processing in a local computer, the user only needs to check the corresponding checkbox and set the number of cores (the default value is number of available cores minus one).

#### Utilization of Distributed Computing

We modified the essential code of CT-FIRE and CurveAlign to make them as headless executables that can run in a distributed computing environment. The parameter settings can be passed to the process functions in the form of text files instead of graphical interface. Five running modes were set, which allows CT-FIRE and CurveAlign as well as some ROI analysis functions can run separately or sequentially. The MATLAB code was compiled in the compilation node with Linux MATLAB support through the server running by the Center for High Throughput Computing (CHTC) at the University of Wisconsin-Madison. Users are provided with a workflow with all the necessary executable files as well as the templates to set both the submission file and the fiber analysis parameter files to run on the CHTC servers.

As a test case, we used the HTCondor-based (Thain et al., 2005) cluster servers. As the tasks of fiber quantification can be allocated to different computers running independently, the computational time can ideally be reduced to 1/*N* (number of computers used in the cluster node set in a job submission file) of the convention computation time when the tool runs on a single computer without enabling multi-core computing. For example, we had run the CHTC workflow on a breast cancer dataset containing 5726 images with a size of 1024 × 1024 pixels for each image, and the dataset was assigned to 287 jobs with each job processing up to 20 images. The 287 jobs run simultaneously, and most jobs took 1–2 h to complete with a few taking up to ∼9 h. However, if running this workload on a single core sequentially, assuming that each image takes 0.1 h to process, it would take more than 500 h.

### ROI Analysis

Full image analysis can be time-consuming and may lack the specific information of some ROIs such as the regions of tumor cells or cells cluster. To quantify the collagen in given regions, ROI module was developed that provides the user with operations for annotating/saving/deleting/loading/analyzing ROIs of any arbitrary or user-specified shapes. Automatic ROI generation code was also developed, and the computed ROI files can be loaded into the tools. In addition, intensity calculation on or within or outside specified ROI(s) was also developed based on the Tumor Trace tool (Pehlke, 2012). This intensity measure can be in combination with other measures such as alignment and fiber density for a better tumor or cell characterization. For use in tumors, high collagen concentrations result in an increased rate of tumor progression. Intensity can be used to identify areas of high-density collagen. Intensity around the outline of an ROI can be used to determine if invasive clusters remodeled collagen differently from non-invasive clusters. When combined with orientation, high-intensity values can be used to detect areas where compounds attract collagen together. Analyzing collagen in this way can help understand relationships of collagen and different compounds, which can be used to identify agents capable of shrinking tumors, and concepts such as the epithelial–stromal relationship. In the operating room, collagen density and collagen fiber alignment can be used to determine the extent to which a tumor has been removed, or in a pathology lab to determine the patient prognosis and help find the best therapeutic approach.

To use the ROI analysis module, the user can first launch the ROI manager module to prepare the ROI annotations or load available ROI files. In ROI management, in addition to ROI annotation, the user can also do ROI analysis for one or more selected ROIs in a single image. A separate ROI analysis module was designed to run specified ROI analysis on all the defined ROIs of each input image. Figures 2, 3 show the analysis of rectangular ROIs.

### Pre- and Post-processing Modules

In CT-FIRE, a post-processing module was implemented to enable the user to visualize individual fibers and check their properties based on the fiber labels. Four different thresholds based on absolute value, percentage, top and bottom number of fibers for length, width, straightness, and angle were developed and can be used to select fibers of interest. The threshold can be applied to one or a combination of two or more fiber properties. For instance, while we can select the 10 longest fibers, we can also select the fibers whose length is longer than 100 pixels and average width is thicker than 8 pixels. If no threshold is applied, this module can merely be used as a tool to combine the separate output files into one excel sheet and combine the summary statistics of each image including median, mode, mean, variance, standard deviation, minimum, maximum, etc.

In CurveAlign, the post-processing module was developed to combine the output files of all the images in the output folder including the summary statistics of the fiber features and the features of curvelets or individual fibers.

In distributed computing applications, a separate MATLAB GUI was developed to enable the user to prepare job files that can be recognized by the job submission file in the CHTC server and unzip the output files from CHTC server into a same folder. Depending on the analysis mode, the user can compress image file or a combination of image file and ROI folder/CT-FIRE output folder/tumor boundary folder into a compressed job file.

## Results

The description of the images processed here can be found in Section “Materials and Equipment.”

### Validation Using Synthetic Fiber Generator

In CT-FIRE, the background threshold was set to 60. In CurveAlign CT mode analysis, 0.35% largest coefficients at the second finest scale were selected and the curvelets group radius was changed from the default of 10 pixels to 8 pixels. Default values were used for other running parameters in the validation. Both CT-FIRE mode and CT mode were tested for all the images in each dataset. The orientation measurement is the most concerned features in the applications on which the most important features extracted from our tools are based including the relative alignment to the tumor boundary and the relative alignment to each other as indicated in TACS models. A two-sided *t* test was used to test whether the calculated overall orientation and alignment equal to the specified values, i.e., 90° and 0.2, respectively. The level of significance was set at *p* < 0.05.

**FIGURE 1:**
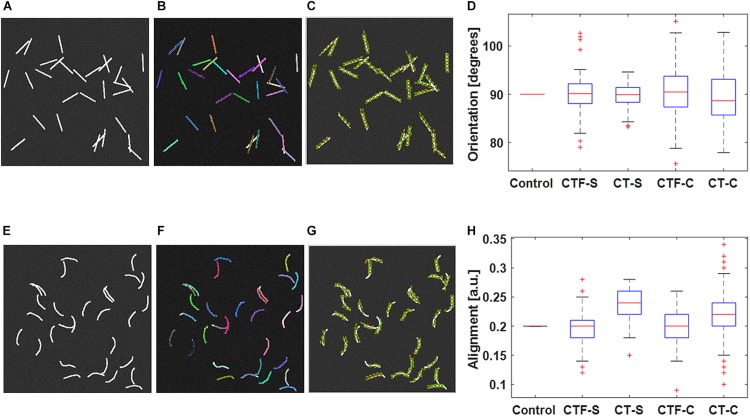
Validation of individual fiber tracking and orientation detection. **(A)** shows the 30 straight fibers with given fiber properties. **(B)** CT-FIRE individual fiber overlay image of **(A)**. **(C)** CurveAlign CT-curvelets overlay image of **(A)**. **(E)** shows the 30 curvy fibers with given fiber properties. **(F)** CT-FIRE individual fiber overlay image of **(E)**; CurveAlign CT-curvelets overlay image of **(A)**. Both CT-FIRE fibers [color lines in **(B)** and **(F)**] and curvelets [green lines with red center point in **(C)** and **(G)**] are mostly faithfully overlaid on the actual fiber directions. The box plots of average orientation and alignment of the 100 synthetic images in each dataset are shown in **(D)** and **(H)**, respectively. The box plots **(D)** and **(H)** of the 100 synthetic images in each dataset show that the calculated orientations and alignment are close to the setting parameters. In the boxplot, the red line represents the median; the blue lines represent the 25th and 75th percentiles, respectively; the dashed lines and black lines indicate the lower and upper limits of the data points that are not considered as outliers; and the red crosses represent outliers. The *t* test shows that all the analysis modes yield the same mean values as the control values for both orientation and alignment at the 5% significance level except for the alignment calculated under the CT mode. CTF-S, CT-FIRE individual fiber mode for straight fiber images; CT-S, CT-curvelets mode for straight fiber images; CTF-C, CT-FIRE individual fiber mode for curvy fiber images; CT-C, CT-curvelets mode for curvy fiber images.

Figure 1 shows validation results of the fiber tracking or orientation detection. The figure shows that both CT-FIRE fibers and curvelets are mostly faithfully overlaid on the actual fiber directions. The box plots show that the calculated orientations and alignment are close to the setting parameters. The *t* test shows that all the analysis modes yield same mean values as the control values for the orientation. The CT-FIRE fiber mode yielded the same values as the control value for the alignment coefficient but the alignments calculated under the CT mode have a bias to the setting value. This is largely due to the fact that the alignment from CT mode is calculated from curvelets and not from the individual fibers that were used to define the alignment in the fiber generator. We noticed that the CT mode usually generates much more fiber directions (same directions if fiber is straight or different directions if fiber is curvy) than single fiber mode, which may result in usually different alignment calculation and possible different orientations (e.g., if fiber length is not equal or fiber is curvy) between these two analysis modes.

**FIGURE 2:**
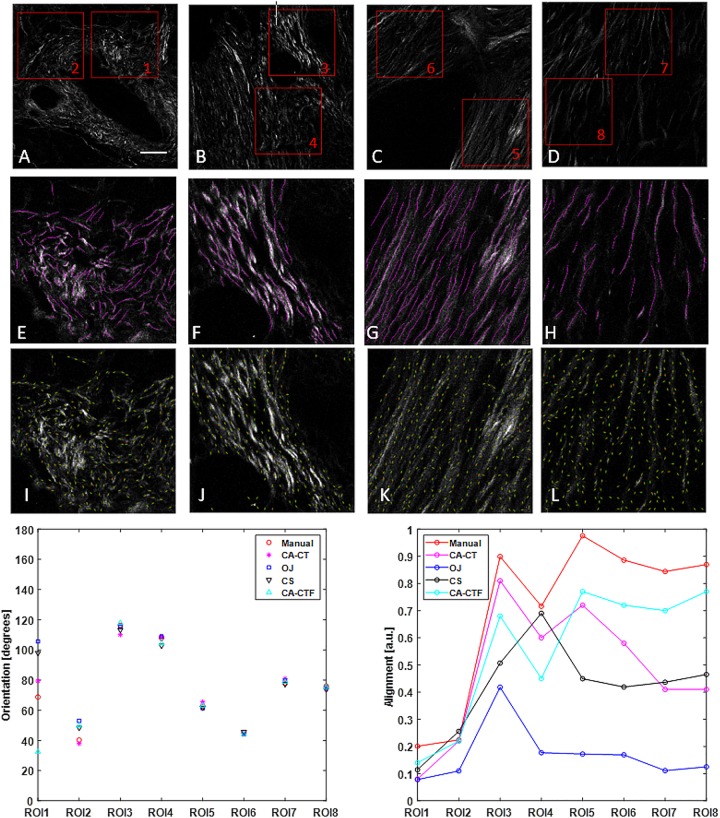
Collagen orientation calculation on SHG images of human pancreatic tissue with CurveAlign (CA) and its comparison with manual measurement and two other open-source tools including structure tenor based OrientationJ (OJ) and Fourier transform-based CytoSpectre (CS). **(A–D)** show the original SHG images and the location of eight ROIs. **(E–H)** show the manually labeled fibers (in magenta) overlaid on the original images of ROIs 1, 3, 5, and 7, respectively. **(I–L)** show curvelets (in green color) represented orientations on the original images of ROIs 1, 3, 5 and 7, respectively. The bottom row shows the comparison in orientation (left) and alignment (right). For the ROIs with relative larger alignment coefficient including ROIs 3–8, all the methods yield similar orientation. For ROIs 1–2 with relative small alignment, the discrepancy becomes bigger, with the CurveAlign Curvelets analysis mode (CA-CT) being closest to the manual measurement. All the methods share some similar trends in alignment measurement, with the CurveAlign fiber segments analysis mode (CA-CTF) being closest to the manual measurement. The differences are mainly due to the different fiber orientation detection algorithm and fiber alignment definition. Images **(A–D)** are shown at the same scale while images **(E–L)** are shown at the same scale. Scale bar equals 50 microns.

**FIGURE 3:**
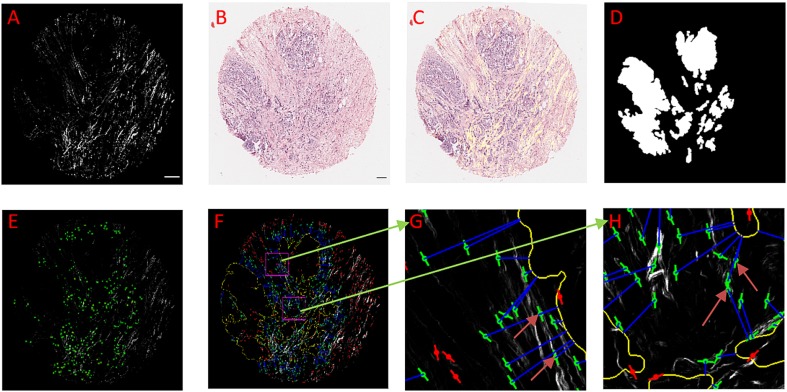
An example of using CurveAlign to create tumor boundary from a bright-field image and quantify the relative angle on a breast cancer TMA core. **(A)** The SHG image, **(B)** Original bright-field image, **(C)** SHG image (in yellow) overlaid on top of the registered bright-field image. **(D)** Segmentation generated boundary mask. **(E)** Heatmap of the relative angle with red color shows the region where one or more fibers have an angle larger than 60°. **(F)** CurveAlign overlay image with the lines indicating the fiber center locations and orientations overlaid on the top of the SHG image; the outline of the tumor boundary is highlighted in yellow, and the two rectangular ROIs are in magenta; a blue line is used to associate the center of fiber with the corresponding boundary locations, and the red lines indicate the fibers located either beyond the distance range or inside the boundary, and green lines are the fibers of interest. **(G)** and **(H)** Zoomed-in ROI results. The arrows in these images show that the fibers either more parallelly aligned in **(G)** with respect to the boundary or are more perpendicularly aligned in **(H)** with respect to the tumor boundary. In boundary creation, pixel per micron ratio was set to 1.65; fiber extraction in CT-FIRE used default settings; the distance parameter was set to 150 pixels; the SHG image size is 2048 by 2048 pixels, and the sizes of ROI are identically set to 256 by 256 pixels. Images **(A,C–F)** are shown at the same scale while images **(G)** and **(H)** are shown at the same scale. Scale bar equals 100 microns.

In other tests on the individual fiber extraction, the CT-FIRE-extracted fibers yielded a larger fiber width and smaller overall fiber length. This might mainly be due to the error of the fiber extract algorithm to resolve the joint points and fiber end points as well as the approximation of the width calculation method used. As it is beyond the scope of this paper, we do not provide details here.

### Comparison of Fiber Orientation Calculation on Pancreatic Tissue Images

Figure 2 shows the comparison of the fiber orientation calculation between our method and three other quantitative approaches. The four images used here were taken from slides of human pancreatic tissue. Two ROIs were annotated in each image and the size of each ROI is 256 × 256 pixels. CurveAlign CT mode and fiber segments mode were applied to the 8 ROIs to calculate the orientation and alignment of each ROI. The CT-FIRE analysis used the default running parameters with the exception of setting fiber length threshold to 15 pixels (30 by default). The threshold of the remaining CT coefficients was set to 20%. For the manual measurement, ImageJ plugin NeuronJ (Meijering et al., 2004) was used to assist the fiber annotation and record the fiber locations and fiber length. A MATLAB script was used to calculate the fiber angle based on the coordinates of two ends. The orientation was calculated from the fiber angles that are weighted against the fiber lengths. The alignment and orientation of manual annotations have the same definition as those in CurveAlign. OrientationJ and CytoSpectre use different definitions to characterize the anisotropy of fiber orientations compared to CurveAlign. In Figure 2, the alignment for OrientationJ uses the coherency calculated in the tool, while the alignment for CytoSpectre equals 1 - circular variance of the orientations. Default parameters were used to run both OrientationJ and CytoSpectre.

The comparison shows that in the orientation calculation, all the methods yielded similar results except for first two ROIs where the CurveAlign curvelets analysis mode is closest to the manual measurement; in the alignment calculation, it is expected that they are different as they are based on different interpretations of individual orientations, but they share similar alignment trends with the CurveAlign fiber segment analysis mode being closest to the manual measurement.

### Example Application on Human Breast Cancer Diagnosis

Figure 3 shows tumor boundary creation from bright-field image of a TACS-3 positive breast cancer H&E slide and SHG image as well as the relative angle measurement. CT-FIRE ran first with the default parameters, and then CurveAlign imported the individual fibers output CT-FIRE and loaded the tiff boundary generated by the boundary creation module with the distance threshold set to 150 pixels. The figure shows that the bright-field image is almost perfectly overlaid on the SHG image, which indicates a successful image registration. The segmented boundaries agree with real boundaries in virtual inspection. The heatmap shows some potential TACS-3-positive regions highlighted in red color. The zoomed-in regions show a non-TACS region with all the fibers parallelly oriented toward the boundary and a TACS region with some of the fibers perpendicularly oriented toward the boundary.

### Example Application on a Mouse Breast Cancer Model

**FIGURE 4:**
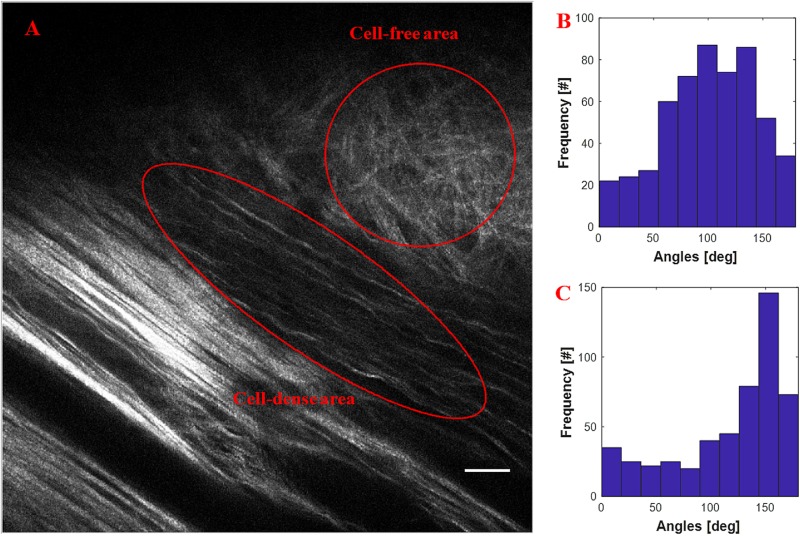
An example of quantification of invading cells reorganizing the ECM of an adult mouse implanted with xenograft of MDA-231 breast cancer cells for 3 weeks. **(A)** SHG image showing the boundary of tumor cell/collagen. **(B)** Histogram of fiber angles in the cell-free area of collagen. **(C)** Histogram of fiber angles in the cell-dense area of collagen. The histograms indicate that the collagen fibers are more aligned in the cell-dense area than the fibers in the cell-free area. Scale bar equals 25 microns.

Figure 4 shows the quantification of collagen fiber alignment in a cell-free area and a cell-dense area in a mouse breast cancer model, respectively. The two ROIs were annotated in Fiji software and then loaded into the CurveAlign “ROI Manager” module. The CurveAlign curvelets mode was first used to analyze the whole image with the threshold of the remaining CT coefficients set to 8%. ROI analysis was then conducted in CurveAlign software to extract the fiber angles within each ROI. Histograms in Figures 4B,C indicate that collagen fibers in invading cell-dense area are more aligned than those in the invading cell-free area. To be noted, the presence of highly aligned collagen fibers in regions with high cell density, suggesting organization of this matrix by tumor cells, is in contrast to acellular regions containing a random distribution of collagen fibers. Thus, when combining multiphoton excitation of endogenous or exogenous fluorophores to image cells with SHG to image the matrix, quantitative information can be obtained to relate these critical patterns to cell organization and phenotype to help facilitate quantitative mapping of the heterogeneous tumor mass.

## Discussion

CurveAlign CT mode requires that the user sets what percentage of the largest curvelet coefficients will be used in an analysis. The selection of this threshold can have an impact on the resulting measurements; however, as indicated in our tests not shown here, there is often little effect on the shape of the resulting fiber angle distribution as the threshold is adjusted. The image is thresholded by the user’s selection of a coefficient threshold with the largest coefficients corresponding to the highest frequency edges in the image, which, in the case of a collagen SHG image, are the strongest and most defined fibers. The larger the threshold value, the greater the number of fibers that will be analyzed. To be noted, if the collagen intensity has a big variation within an image, only the brightest collagen fibers can be detected by CT mode. To do an accurate calculation, ROI may need to be defined to ensure that collagen fibers are the dominated signal and do not have apparent intensity variation.

The CT uses angled polar wedges or angled trapezoid windows in frequency domain to provide higher directional selectivity than both the Fourier transform and the conventional wavelet transform (Ma and Plonka, 2010). Fourier analysis usually works well on periodic structures, and wavelet analysis works well on point singularities; however, neither is well suited for the task of sparsely representing line-like edges. To be noted, the Fourier filter extracts oriented patterns at all scales whereas the curvelets are sensitive to oriented patterns at different scales and can increase directional selectivity at fine scales according to parabolic scaling law. Hence, CT is well suited to the analysis and synthesis of images with highly directional features (Püspöki et al., 2016).

In the comparison on orientation calculation between our tool and three other quantitative approaches as shown in Figure 2, large differences were observed when fibers are more randomly oriented, for example in ROIs 1 and 2. Besides the accuracy of each method compared, another reason might be due to the observations that the ability of some methods including the Fourier transform-based method to measure principal angle tends to decrease as the anisotropy of orientations or alignment becomes small (Sander and Barocas, 2009). As for the orientation calculation by our tool, in practice, the user can rely on virtual inspection to empirically verify the tracing results.

In the C++ implementation for fast individual fiber extraction, although the MATLAB code was used as a reference to validate the C++ MEX code, the C++ MEX functions have some steps implemented differently from the MATLAB code and thus identical fiber extraction results between the C++ MEX code and the original MATLAB code should not be expected. The current MEX code can be used to estimate individual fiber parameters in a much faster manner than the original code and should yield similar fiber extraction for images with clear fiber presences as indicated in our tests on synthetic fibers. However, the current code cannot fill gaps between fiber segments or fiber branches, which may lead to mis-segmented long fibers when intensity changes along a fiber direction. The ongoing efforts lie on tackling this issue, optimizing other necessary steps in C++ with the long-term goal of integrating the C++ fast CT-FIRE code into image acquisition software for real-time fiber estimation.

The synthetic fiber generator software tool has many useful features. One of the remaining challenges is to better represent the interactions between two fibers that have joint points. The information of joint points is particularly useful when tissue mechanical properties are concerned. Another work on the list is to automatically load the fiber parameters calculated from a large dataset to generate more realistic, large scale synthetic fiber image datasets that could be potentially used for machine learning and other purposes. The current version can be run in a non-interactive, command-line only mode by passing the parameter settings through a standard JavaScript Object Notation (JSON) data interchange file. One planned and straightforward way for an automatic fiber generation would be to extract information from the CT-FIRE/CurveAlign output files to automatically update the JSON file.

The CHTC workflow for large-scale fiber quantification has been tested and used successfully in some real projects including a prostate cancer dataset and a breast cancer dataset each consisting of thousands of images. To be noted, for the distributed computing systems, the job files need to be carefully configured to fully use the computing power while reducing the time of file pre-/post-processing and file transfer to and from the server.

Heatmaps have been created for relative angle and a local alignment feature that can facilitate the detection of ROIs. We are planning to create heatmaps for all the individual fiber properties as well as the localized fiber density and alignment features. Commonly used statistical analysis such as Student’s *t* test, boxplot, clustering, and classification are hopefully added to our fibrillar collagen quantification workflow to gain valuable insights from the output features.

To take advantage of the ecosystem of ImageJ/Fiji (Schindelin et al., 2012; Schneider et al., 2012) and other SciJava-compatible software systems being developed by our lab, an ImageJ OPs (Rueden et al., 2017) for collagen orientation and alignment analysis based on CT is under development. This OPs will be part of a future comprehensive workflow that can integrate the information from other OPs and plug-ins, such as automatic segmentation, cell measurement, ROI management, etc. Moreover, we have developed a prototype of KNIME (Fillbrunn et al., 2017) nodes for CurveAlign and CT-FIRE for a convenient feature statistical analysis, classification, and visualization. These are all planned future additions for new releases of the CurveAlign/CT-FFIRE platform.

In fibrillar collagen quantification, although we have applications of using collagen fiber geometry properties such as fiber width or thickness (LeBert et al., 2015) and fiber length (Hanley et al., 2016), the collagen fiber alignment is the most commonly used and robust measure in our applications (Bredfeldt et al., 2014a; Drifka et al., 2015; Conklin et al., 2018). This is partially because there are no mature research models in thickness or length characterization like the TACS model (Provenzano et al., 2006; Conklin et al., 2011) in alignment. Moreover, technically, there are challenges to accurately calculate the thickness and length if the fibers are close to each other, have joints, have significant intensity changes along their propagation directions, etc. In addition, for the SHG imaging of collagen, the width and length of the fiber are largely numeric aperture (NA) dependent and we cannot know conclusively if the length or width belong to a single fibril, fiber, or a bundle of fibers. However, orientation and alignment are much less affected by the NA. In our experience, to resolve a robust thickness measurement in SHG imaging, the objective lens should usually be 40× or higher with NA ≥ 1.0. A long collagen fiber may be divided into smaller segments if the fiber is curvy and the intensity or thickness along the fiber is varying or it has joints with other fibers. In the individual fiber extraction algorithm, a more accurate width at each nucleation point can be calculated by improving the edge detection and taking into account the distance to both fiber edges instead of using the distance to one edge as the fiber radius. To improve the accuracy of fiber length calculation, introducing the local orientation or anisotropy information based on other available methods (Meijering et al., 2004; Rubbens et al., 2009; Rezakhaniha et al., 2012; Kabir et al., 2013; Quinn and Georgakoudi, 2013; Boudaoud et al., 2014; Sun et al., 2015) into the fiber extraction algorithm may help form an intact fiber. In addition, we can also try adapting energy minimization (Mori and van Zijl, 2002) along the fiber direction to the current algorithm.

Though the current version of our tools can be merely used for 2-D image analysis, both the CT and individual fiber extraction algorithms have 3-D implementations and our tools could adapt them if there are real needs. However, the commonly used collagen imaging modalities either do not resolve sufficient depth information (e.g., polarized light microscopy) or do not often have good enough axial resolution (e.g., SHG) for 3D fiber measurement. 3D fluorescence microscopy collagen images are reported but have limited uses as yet. We will implement 3D fiber analysis support as imaging methods for such 3D analysis evolve and become available.

## Conclusion

In summary, we developed a powerful, comprehensive fibrillar collagen quantification platform based on the CT. This platform can meet crucial needs from both the basic science research and the clinical studies, and has been actively used by ourselves and other researchers across the world for a wide array of applications. We have been putting continuous efforts on getting the tools ready for more and more new applications with a future focus on improved performance and interoperability.

## Data Availability Statement

The data utilized in this study are available from the corresponding author, (KE), upon request.

## Author Contributions

YL, AK, CP, JB, and KE conceptualized and designed the study. All authors contributed to the experiments and to descriptions in the manuscript.

## Conflict of Interest

The authors declare that the research was conducted in the absence of any commercial or financial relationships that could be construed as a potential conflict of interest.

## Footnotes

1 MEX files are external functions that can be written in a language like C++ and called from MATLAB.
