# Supplementary material for: Fibrillar Collagen Quantification With Curvelet Transform Based Computational Methods
Source: Front Bioeng Biotechnol. 2020 Apr 21;8:198. doi: 10.3389/fbioe.2020.00198 (PMC7186312; doi:10.3389/fbioe.2020.00198)
Supplement: Supplementary file 1 [file Data_Sheet_1.docx]

**Supplementary Figure 1**. A speed test on both synthetic images (upper row) and real images (lower row) shows that MEX functions are more than 100x faster (left) than the original code to complete three steps of fiber extraction from single images while leading to similar orientation(middle) and alignment (right). The texts in the left column are the mean value ± standard deviation (sample number). Test images include 100 synthetic straight fiber images and 4 real SHG images of pancreatic cancer samples described in section 4.1 and section 4.2, respectively. Both the original code and the fast fiber extraction code with the modified MEX functions were applied to the test data sets. The running parameters for the synthetic fibers are the same as those described in section 4.1. For the 4 real images, they used the same parameters of the 8 ROIs described in section 4.2. The test was conducted on a Windows computer with AMD FX(tm)-8300 8-core processor, 32 GB memory with Windows 10 64-bit operating system and MATLAB 2018b. In the boxplot, the red line represents the median, the blue lines represent the 25th and 75th percentiles, respectively, the dashed lines and black lines indicate the lower and upper limits of the data points that are not considered as outliers, and the red crosses represent outliers. MEX-SYN: MEX functions applied on synthetic images; ORI-SYN: corresponding original code applied on the synthetic images; MEX-Real: MEX functions applied on real images; ORI-Real: corresponding original code applied on real images.
