# Supplementary figures and images for: Fibrillar Collagen Quantification With Curvelet Transform Based Computational Methods
Source: Front Bioeng Biotechnol. 2020 Apr 21;8:198. doi: 10.3389/fbioe.2020.00198 (PMC7186312; doi:10.3389/fbioe.2020.00198)

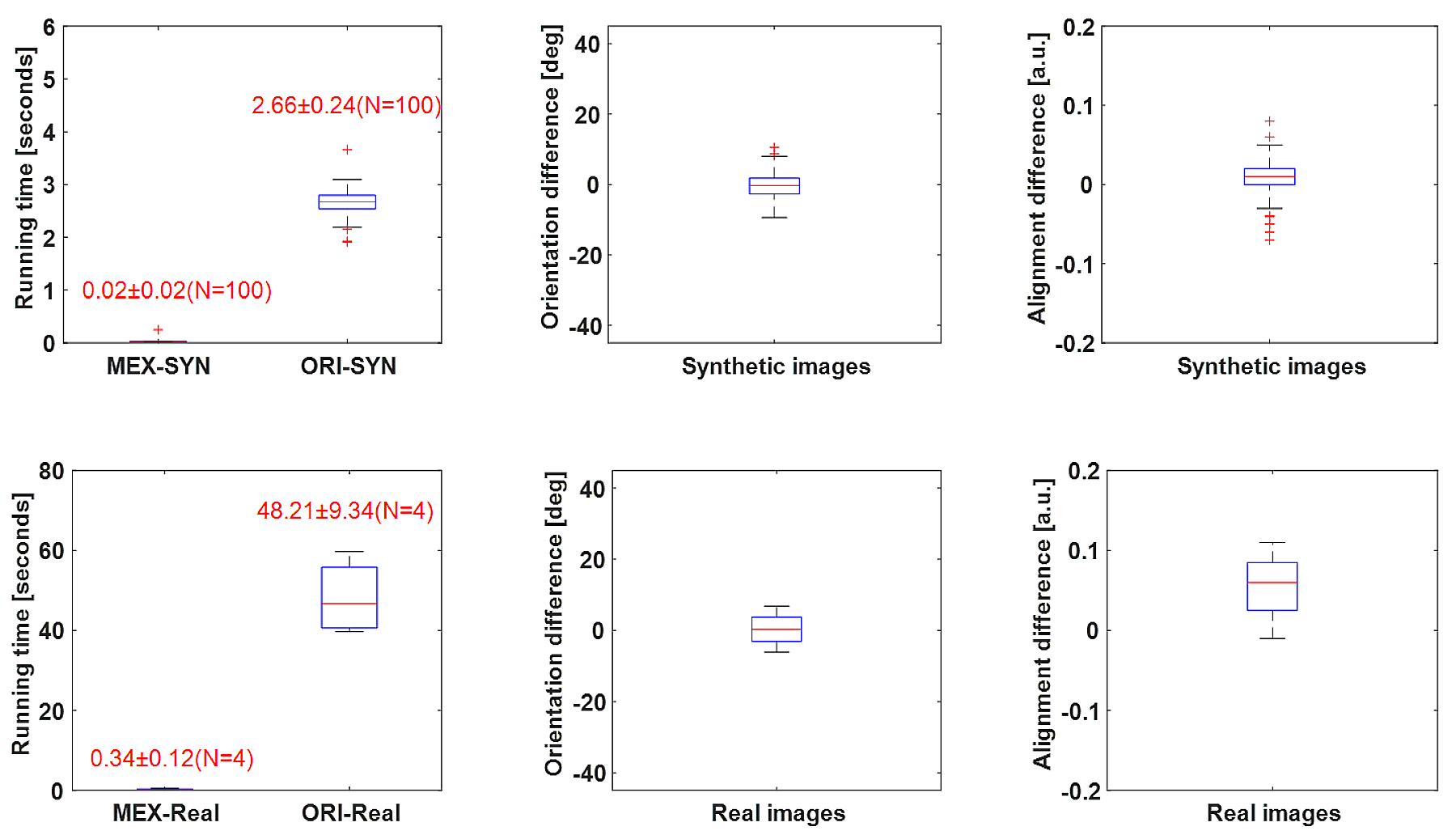

Supplement: Supplementary file 2 [file Image_1.TIF]
